# Long-Term Impact of Community-Based Information, Education and Communication Activities on Food Hygiene and Food Safety Behaviors in Vietnam: A Longitudinal Study

**DOI:** 10.1371/journal.pone.0070654

**Published:** 2013-08-12

**Authors:** Kumiko Takanashi, Dao To Quyen, Nguyen Thi Le Hoa, Nguyen Cong Khan, Junko Yasuoka, Masamine Jimba

**Affiliations:** 1 Department of Community and Global Health, Graduate School of Medicine, The University of Tokyo, Tokyo, Japan; 2 International Life Sciences Institute Japan Center for Health Promotion, Tokyo, Japan; 3 Department of Food Science and Safety, National Institute of Nutrition, Hanoi, Vietnam; 4 Department of Science and Training Management, Ministry of Health, Hanoi, Vietnam; Iran University of Medical Sciences, Iran

## Abstract

**Background:**

Ingestion of contaminated water or food is a major contributor to childhood diarrhea in developing countries. In Vietnam, the use of community-based information, education and communication (IEC) activities could be a sustainable strategy to improve food hygiene and food safety behaviors. This study thus examined the long-term impact of community-based IEC activities on food hygiene and food safety behaviors.

**Methods:**

In this longitudinal study, we interviewed caregivers of children aged between six months and four years in suburban Hanoi. Baseline data were collected in January 2006 (n = 125). After conducting IEC interventions, we collected a 1^st^ set of evaluation data in January 2007 (n = 132). To examine the long-term impact of the interventions, we then collected a 2^nd^ set of evaluation data in January 2008 (n = 185). Changes in childhood diarrhea prevalence, IEC coverage, and food hygiene and food safety behaviors were assessed over a two-year period using bivariate and logistic regression analyses. Effective IEC channels were determined through multiple linear regression analysis.

**Results:**

Childhood diarrhea was significantly reduced from 21.6% at baseline to 7.6% at the 1^st^ post-intervention evaluation (P = 0.002), and to 5.9% at the 2^nd^ evaluation. Among 17 food hygiene and food safety behaviors measured, a total of 11 behaviors were improved or maintained by the 2^nd^ evaluation. Handwashing after toilet use was significantly improved at both evaluation points. Overall, 3 food safety behaviors and 7 food hygiene behaviors were found to have significantly improved at the 1^st^ and at the 2^nd^ evaluations, respectively. Flip chart communication administered by community groups was identified to be the most effective IEC channel for effecting behavior change (P = 0.018).

**Conclusions:**

Flip chart communication administered by community groups is effective for improving multiple food hygiene and food safety behaviors in sustainable ways, and should be included in water and health promotion programs.

## Background

In developing countries, diarrheal diseases remain the second leading cause of death among children under 5 years [1]. The main biological causes of childhood diarrhea include ingesting contaminated water or food, and transmission of pathogens from contaminated hands [2], [3].

Food hygiene and food safety (FHFS) promotion has been identified as an effective measure to prevent fecal-oral pathogen transmission [4]. In this vein, simple hygiene behaviors such as handwashing are the most recommended interventions worldwide [5]. Specifically, the five critical handwashing points commonly cited are *before eating*, *before feeding children*, *before preparing food*, *after using the toilet*, and *after cleaning a child's bottom* [6]. Basic food safety interventions are also recognized as important in disrupting gastrointestinal pathogen transmission and growth in food [7]. Five major control factors in this area include *personal hygiene*, *adequate cooking*, *avoiding cross-contamination*, *keeping food at a safe temperature* and *avoiding foods from unsafe sources* [8]. On these principles, FHFS interventions can thus be expected to significantly reduce childhood diarrhea [4], [9].

Information, Education and Communication (IEC) interventions have been employed using various methods to improve FHFS behaviors [10], but little is known about their long-term effectiveness [11]. Traditionally, the IEC strategy involves targeting a small number of behaviors using only a few core messages, repeating those messages through several information channels [12] in order to overcome the weaknesses inherent in individual approaches [13]. This strategy tends to achieve higher rates of adherence to targeted behaviors [14]. So far, long-term sustainability of improved behaviors has been demonstrated for interventions targeting only a limited number of handwashing behaviors [15], [16]. A few studies have also targeted FHFS behaviors, but still focused on a limited range of behaviors and examined only medium-term (3–5 months) sustainability [17], [18]. Only one study, meanwhile, has demonstrated the long-term (2-year) effectiveness of community-based IEC interventions on multiple behaviors – and it did not cover food safety behaviors [19]. Notably, the adherence rate in this study was comparable to studies targeting only a small number of behaviors. To date, we know of no studies that have aimed to improve a large number of FHFS behaviors while also examining the long-term effectiveness of IEC interventions.

In Vietnam, socioeconomic status, lack of piped water and latrines, less frequent handwashing [20] and contaminated food have been identified as potential contributing factors to the incidence of diarrheal diseases [21], [22]. Several different government institutions within the country address these issues collaboratively using IEC strategies [23]. The most common such approaches include health day events and utilize traditional communication channels such as music, poetry and theater [24]. Such activities have the potential to influence water- and health-related awareness and behaviors.

However, these common IEC approaches have several shortcomings [25]. First, didactic IEC approaches promote information and education through a single channel. Second, IEC messages and materials do not adequately reach community members due to their inherently top-down approach. Finally, even IEC approaches that improve knowledge about water- and health-related subjects do not always change the associated behaviors. Therefore, an innovative approach is needed for community-based IEC activities in order to promote and maintain FHFS behaviors in Vietnam.

We designed this study within the Safe Water and Nutrition (SWAN) Project run by the International Life Sciences Institute Japan Center for Health Promotion (ILSI Japan CHP) and the National Institute of Nutrition (NIN) in Hanoi (File S1). This community-based project renovated the infrastructure of a water treatment facility (WTF) to improve water quality and quantity, and conducted operation and maintenance training for the water management union (WMU). Within this project, an IEC program was implemented toward improving behaviors related to drinking water, FHFS and nutrition. In this context, our study examined the long-term impact of community-based IEC activities on FHFS behaviors.

## Methods

### Study site

We conducted this longitudinal study in Huynh Cung Village, Tam Hiep Commune in the Thanh Tri District of Hanoi, Vietnam – home to 3,900 people in 2006 [22]. Located immediately south of Hanoi, this suburban district tends to be an outlet for accumulated urban waste carried by water flowing in streams and canals from Hanoi. Nevertheless, community members generally have enough water for their daily requirements throughout the year [26]. Traditionally they have only consumed tube well water and rain water after boiling. A WTF exists in the village, but only two-thirds of community members use WTF water through piped supplies. Close relationships with relatives and neighbors at the community level provide an environment where caregivers can discuss FHFS issues without any negative social repercussions.

### Study population

This study targeted caregivers and their children aged 6 months to 4 years. The term “caregivers” is used throughout this paper to refer collectively to parents or other family members who are responsible for a child's day-to-day primary care and upbringing. From the list of children under 5 years maintained at the commune health station, we identified 298 caregiver-child pairs for the baseline survey, 320 for the 1^st^ evaluation survey and 356 for the 2^nd^ evaluation survey. Among these, 220 caregiver-child pairs were enrolled at the time of the baseline survey, 208 at the time of the 1^st^ evaluation survey and 274 at the time of the 2^nd^ evaluation survey. The main reasons for non-participation were family obligations and sudden illness. From the enrolled caregiver-child pairs, we excluded from the statistical analyses those caregivers who did not receive WTF water, and those who were not the children's primary caregivers.

We calculated the required sample size to detect a 25% difference in FHFS behaviors from the baseline survey to the 1^st^ evaluation survey, with a confidence interval of 95% and a power of 80% (Epi Info 3.5.3.). This entailed a minimum of 120 caregiver-child pairs in each survey.

### Data collection

Eight to ten trained interviewers conducted interviews at the commune health center in January 2006 (baseline), January 2007 (1^st^ evaluation) and January 2008 (2^nd^ evaluation). Before each of the three survey waves, the same Vietnamese researcher explained the details of each question to the interviewers to ensure accurate data collection.

### Questionnaire development

A structured questionnaire was developed for this study. First, we modified a questionnaire used in a similar survey carried out by Vietnam's Ministry of Health [27], [28]. Then, we added FHFS questions related to WHO recommendations [8], [29]. Finally, we incorporated ideas gathered during group discussions with the caregivers. The final questionnaire covered socio-demographic characteristics, water use, prevalence of childhood diarrhea, and FHFS behaviors. We added questions about IEC channels to both the 1^st^ and 2^nd^ evaluation surveys. The questionnaire was first developed in English and then translated into Vietnamese by local experts. This version was translated back into English to confirm the accuracy of the original translation. Finally, we tested the Vietnamese questionnaire using 25 caregivers from a different village in the same district.

### Community-based programs (Figure 1)

**Figure 1:**
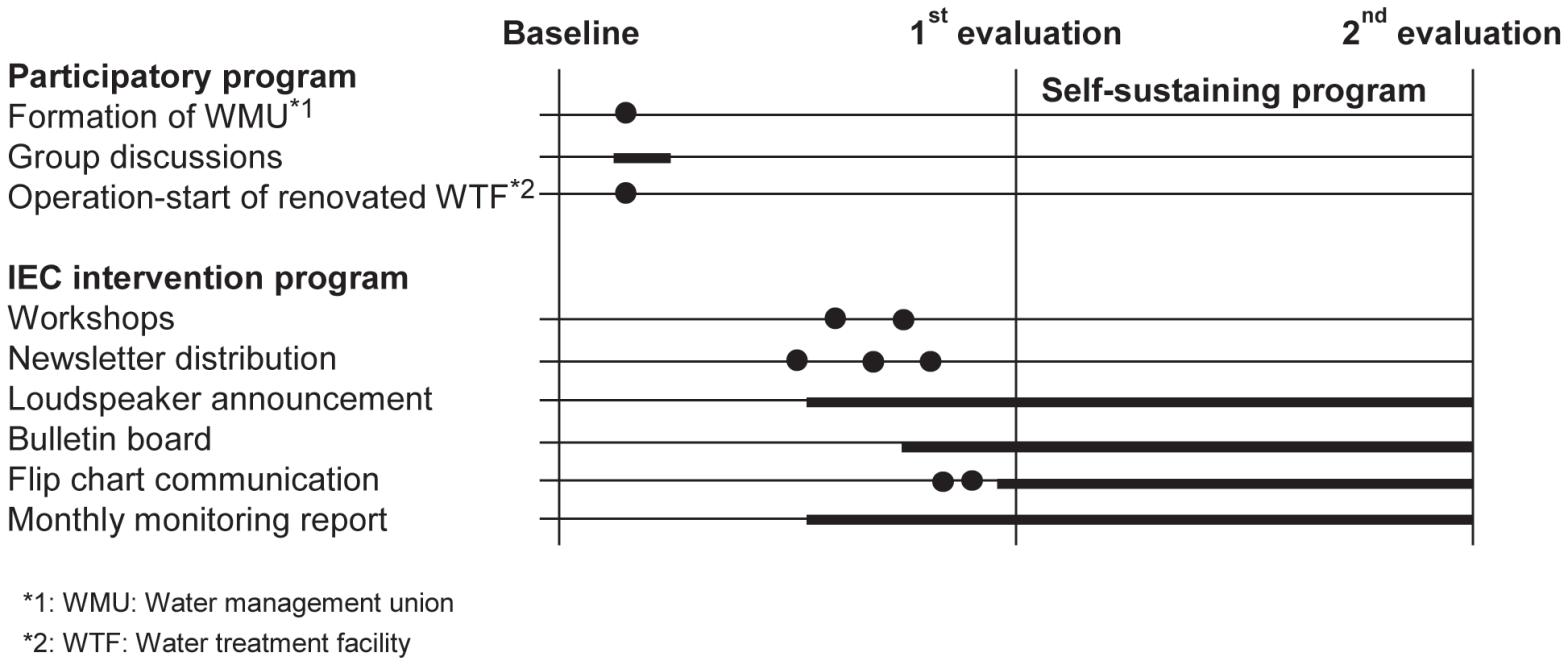
Community-based programs.

#### Participatory program (January to May 2006)

First, we assisted in the formation of a WMU to lead the IEC activities. This WMU consisted of 10 community members: the village leader, sub-group leaders, operators, the first secretaries of the village's communist party, the leader of the health station and village health workers (One sub-group leader serves concurrently as a village health worker and one village health worker serves concurrently as a Women's Union member.). We then selected behaviors related to FHFS based on group discussions with caregivers and the results of the baseline survey. Five IEC channels were also selected through group discussions.

#### IEC intervention program (June to December 2006)

We designed an intervention program to promote behavior change through educational messages linking diarrhea to FHFS behaviors. The main message was, “Both handwashing with soap and proper food handling practices contribute to protecting your child from developing diarrhea.” We also provided 20 pieces of practical advice around such concepts as “washing hands with soap after going to the toilet”, “washing your child's hands with soap before eating”, and “separating utensils for raw and cooked foods” in order to present a clear direction for action.

The WMU provided FHFS messages through five IEC channels: workshops, newsletters, loudspeaker announcements, bulletin boards, and flip chart communication. First, we organized two workshops devoted to FHFS. An estimated 240 caregivers attended each workshop. Second, we issued three newsletters to communicate FHFS-related information. To this end, a Vietnamese journalist interviewed caregivers, local authorities and project teams to select important topics. Third, village health workers wrote articles about FHFS issues and broadcast their messages twice weekly using public loudspeakers (fixed to poles on streets). Fourth, we installed a bulletin board in front of the village cultural center, located on the village's main street, on which the WMU posted the program's FHFS-themed newsletters. Finally, we developed two different flip chart types (6 pages, picture-story style, A3-size in full color) dealing with FHFS issues and water-born diseases, respectively. For each flip chart we conducted a two-day training session, in which the WMU learned how to deliver the main messages effectively using the flip charts and practiced the necessary communication skills through role-play.

#### Self-sustaining IEC program (January 2007 to January 2008)

We designed a self-sustaining program to maintain the WMU's IEC activities and caregivers' FHFS behaviors following the program period. The village health workers continued delivering the loudspeaker announcements twice a week. Similarly, the WMU replaced the materials posted on the bulletin boards periodically, and used nine pairs of flip charts to communicate with caregivers during village gatherings held in the village cultural center and during home visits. The WMU communicated with an average of 35 households every month. Targeted households were exposed to flip chart communication a maximum of two times during this period. One village health worker reported all activities to us in the form of a monthly monitoring report. Additionally, we visited the village to observe on-going activities every two to three months.

### Measurements

Outcome variables included childhood diarrhea prevalence and caregivers' FHFS behaviors. We defined diarrhea as watery stool occurring more than three times per day in the two weeks prior to the survey [30]. In this study, the term “food hygiene” refers to personal hygiene, especially in the form of handwashing with soap at critical points in the course of a day [5]. The term “food safety”, meanwhile, refers to food that does not cause harm when eaten [31]. Independent variables included socio-demographic characteristics, water use details and IEC channels.

We measured 17 FHFS behaviors in total. Ten critical handwashing time points assessed included four during eating and food handling-related activities, four during sanitation-related activities, and children's handwashing before eating and after using the toilet. Seven proper food handling practices assessed consisted of three related to avoiding cross-contamination, three related to keeping food at a safe temperature, and one related to adequate cooking. To evaluate these behaviors as a whole, we developed a scoring system whereby 1 point was awarded for “good behavior” and 0 points for “wrong behavior” on each measured FHFS item. However, we excluded three items from the scoring – “whether or not to reheat leftovers”, “raw food storage” and “cooked food storage” – because we did not measure “whether or not to reheat leftovers” during the baseline survey, none of the caregivers answered incorrectly for “raw food storage” at baseline or at the 2^nd^ evaluation, and none of the caregivers answered incorrectly for “cooked food storage” at the 2^nd^ evaluation. Total scores ranged from 0 to 14. Cronbach's α for the 14-item scale was 0.78 at baseline, 0.64 at the 1^st^ evaluation and 0.59 at the 2^nd^ evaluation.

To examine the effect of multiple IEC channels in this program, we included an item scored as follows. If caregivers reported that they received IEC related to FHFS from any of the IEC channels detailed above, we gave 1 point for each; those who reported “not receiving IEC related to FHFS” received 0 points. Possible scores ranged from 0 to 5.

### Statistical analysis

From the identified caregivers, we analyzed data from 125 caregiver-child pairs (42%) at baseline, 132 (41%) at the 1^st^ evaluation and 185 at the 2^nd^ evaluation (52%). We assessed all categorical variables using the Chi-square test or the Fisher's exact test, and all continuous variables using the student's t-test for changes from the baseline to the 1^st^ evaluation, and from the 1^st^ to the 2^nd^ evaluation. To analyze IEC activities, childhood diarrhea prevalence and FHFS behaviors, after checking for multicollinearity, we performed logistic regression analysis to adjust for confounding factors and reported the adjusted P value. For multiple IEC channels and multiple FHFS behaviors scores, we performed hierarchical multiple regression analysis. Further multiple linear regression with backward elimination procedures was performed to determine the factors affecting the greatest number of good FHFS behaviors. A P value of <0.05 was considered to indicate statistical significance. All statistical analyses were performed using SPSS, version 13.0 (SPSS Inc., Chicago, IL, USA).

### Ethics statement

The Research Ethics Committee of the Graduate School of Medicine of the University of Tokyo, Japan (No. 1329) and the Scientific Committee of the NIN, Vietnam reviewed and approved the study protocol. All the caregivers were informed of the study procedures and voluntarily took part in the study. After explaining the confidentiality of the study, we obtained written informed consent from all caregivers for their participation and that of their children.

## Results

### Socio-demographic characteristics

Only a few of the caregivers' socio-demographic characteristics were statistically different between baseline and the 1^st^ evaluation, and between the 1^st^ and 2^nd^ evaluations (Table 1). In the baseline survey, 96.8% of caregivers were mothers, whereas 77.3% were mothers in the 1^st^ evaluation survey (P<0.001). Occupations were significantly different between the 1^st^ evaluation and 2^nd^ evaluation largely due to an increase in the number of caregivers engaging in home-based businesses in the 2^nd^ evaluation (P = 0.011). In the 1^st^ evaluation, the percentage of caregivers who had large families (5 people or more) was significantly higher than in the baseline survey (P = 0.001), with a similar increase also seen at the 2^nd^ evaluation (P = 0.005). Although variations in children's ages between measurements were not statistically significant, the percentage of children under 24 months was higher in the baseline survey and at the 1^st^ evaluation compared with the 2^nd^ evaluation.

**Table 1:** Caregivers' socio-demographic characteristics.

		Baseline (n = 125)			1^st^ evaluation (n = 132)				2^nd^ evaluation (n = 185)			Baseline to 1^st^ evaluation		1^st^ evaluation to 2^nd^ evaluation		
		n	(%)		n		(%)		n	(%)		P value* ^1^		P value* ^1^		
Caregivers												<0.001		0.230		
Other		4	(3.2)		30		(22.7)		32	(17.3)						
Mother		121	(96.8)		102		(77.3)		153	(82.7)						
Age (years)												0.077		0.681		
29 or younger		49	(39.2)		41		(31.1)		60	(32.4)						
30–34		49	(39.2)		46		(34.8)		56	(30.3)						
35 or older		27	(21.6)		45		(34.1)		69	(37.3)						
Occupation												0.123		0.011		
Farmer		47	(37.6)		46		(34.8)		44	(23.8)						
Factory worker		24	(19.2)		19		(14.4)		27	(14.6)						
Housework/retired		20	(16.0)		37		(28.0)		39	(21.1)						
Civil servant/company employee		14	(11.2)		17		(12.9)		35	(18.9)						
Home-based business* ^2^		20	(16.0)		13		(9.8)		40	(21.6)						
Education												0.622		0.696		
Secondary school or less* ^3^		53	(42.4)		60		(45.5)		80	(43.2)						
High school or more* ^4^		72	(57.6)		72		(54.5)		105	(56.8)						
Number of people in household																
4 or fewer		81	(64.8)		58		(43.9)		111	(60.0)		0.001		0.005		
5 or more		44	(35.2)		74		(56.1)		74	(40.0)						
Refrigerator possession												0.217		0.061		
No		47	(37.6)		40		(30.3)		39	(21.1)						
Yes		78	(62.4)		92		(69.7)		146	(78.9)						
Boil water for drinking																
Yes		124	(99.2)		–		–		–	–						
Sometimes		1	(0.8)		–		–		–	–						
Type of latrine																
No latrine		1	(0.8)		–		–		–	–						
Other type of hygienic latrines* ^5^		23	(18.4)		–		–		–	–						
Water-flush latrine		101	(80.8)		–		–		–	–						
Number of children under five years													0.054		0.446	
1	112			(89.6)	107	(81.1)		156			(84.3)					
2 or more	13			(10.4)	25	(18.9)		29			(15.7)					
Birth order of child* ^6^													0.968		0.542	
Second or higher	59			(47.6)	62	(47.3)		93			(50.8)					
First	65			(52.4)	69	(52.7)		90			(49.2)					
Child's age (months)													0.491		0.084	
6–11	18			(14.4)	13	(9.8)		21			(11.4)					
12–23	35			(28.0)	39	(29.5)		38			(20.5)					
24–35	27			(21.6)	39	(29.5)		42			(22.7)					
36–47	23			(18.4)	23	(17.4)		49			(26.5)					
48–59	22			(17.6)	18	(13.6)		35			(18.9)					
Child's sex																
Male	69			(55.2)	69	(52.3)		103			(55.7)		0.638		0.549	
Female	56			(44.8)	63	(47.7)		82			(44.3)					

* 1: Chi-square test or Fisher's exact test.

* 2: Those with a home-based business include seller, hairdressers, tailors, etc.

* 3: *Secondary school or less* includes not being able to read and write, only being able to read and write, primary school attendance only, and up to secondary school attendance only.

* 4: *High school or more* includes high school and higher education.

* 5: *Other type of hygienic latrines* includes single-vault latrines, double-vault latrines, septic tanks, and biogas-vault latrines (MOH 2005).

*6: Baseline (n = 124), 1^st^ evaluation (n = 131) and 2^nd^ evaluation (n = 183).

### Water use

More than 70% of the caregivers had optimal access to WTF water (more than 60 lit/capita/day) in all three survey waves (Table 2), and more than 80% used WTF water for drinking, cooking, food preparation and washing. Among them, a slightly higher percentage of caregivers tended to use WTF water for food preparation. The percentage of caregivers who used WTF water as their main source of cooking water remained constant between the baseline survey and the 1^st^ evaluation, but slightly increased from 85.6% in the 1^st^ evaluation to 92.4% in the 2^nd^ evaluation.

**Table 2:** Water use situation.

	Baseline (n = 125)			1^st^ evaluation (n = 132)		2^nd^ evaluation (n = 185)		Baseline to 1^st^ evaluation	1^st^ evaluation to 2^nd^ evaluation
	n	(%)	n		(%)	n	(%)	P value* ^1^	P value* ^1^
WTF water access level								0.148	0.471
Basic - Intermediate (0–59 L/c/d)	22	(17.6)	33		(25.0)	53	(28.6)		
Optimal (More than 60 L/c/d)	103	(82.4)	99		(75.0)	132	(71.4)		
Main drinking water source								0.383	0.094
Other water sources* ^2^	16	(12.8)	22		(16.7)	19	(10.3)		
WTF water or Purified bottled water	109	(87.2)	110		(83.2)	166	(89.7)		
Main cooking water source								0.999	0.050
Other water sources* ^2^	18	(14.4)	19		(14.4)	14	(7.6)		
WTF water	107	(85.6)	113		(85.6)	171	(92.4)		
Main food preparation water source								0.735	0.791
Other water sources* ^3^	9	(7.2)	11		(8.3)	17	(9.2)		
WTF water or Purified bottled water	116	(92.8)	121		(91.7)	168	(90.8)		
Main laundry and bathing water source								0.616	0.883
Other water sources* ^3^	18	(14.4)	22		(16.7)	32	(17.3)		
WTF water	107	(85.6)	110		(83.3)	153	(82.7)		

* 1: Chi-square test.

* 2: Rain water or drilled well.

* 3: Rain water, drilled well or dug well.

### Coverage of IEC activities

Overall, IEC channels introduced through the program exhibited higher coverage (67.4–87.1% in the 1^st^ evaluation, 53.0–86.1% in the 2^nd^ evaluation) compared with mass media channels (3.8–28.0% in the 1st evaluation, 10.8–28.1% in the 2^nd^ evaluation) among caregivers in the two surveys (Table 3). The proportion of caregivers who were exposed to radio messages significantly increased from 9.8% in the 1^st^ evaluation to 23.2% in the 2^nd^ evaluation (P = 0.006). Coverage of workshops and newsletters, meanwhile, decreased significantly at the 1^st^ and 2^nd^ evaluation phases because such channels were mainly used prior to the 1^st^ evaluation. Caregivers were exposed to a slightly greater number of the program's IEC channels (3.25 [SD 1.6] channels) at the 2^nd^ evaluation than at the 1^st^ evaluation (3.04 [SD 1.2] channels).

**Table 3:** Coverage of IEC activities.

	1^st^ evaluation (n = 132)		2^nd^ evaluation (n = 185)					
	n	(%)	n	(%)	P value* ^1^	AOR	(95% C.I.)	Adjusted P value* ^2^
**Mass media channel**								
Television	37	(28.0)	52	(28.1)	0.988	0.93	(0.53–1.65)	0.810
Radio	13	(9.8)	43	(23.2)	0.002	2.86	(1.31–6.03)	0.006
Newspaper	5	(3.8)	20	(10.8)	0.022	2.90	(0.91–9.20)	0.071
**Individual IEC channels from the program**								
Attended workshops	91	(68.9)	98	(53.0)	0.004	0.50	(0.29–0.85)	0.010
Read newsletters	106	(80.3)	121	(65.4)	0.004	0.42	(0.23–0.76)	0.004
Heard loudspeaker announcement	115	(87.1)	163	(86.1)	0.792	1.49	(0.68–3.27)	0.324
Saw bulletin board	89	(67.4)	112	(60.5)	0.210	0.73	(0.43–1.24)	0.246
Received flip chart communication	––	–	107	(57.8)	–	–	–	–
**Multiple IEC channels from the program**	Mean	(SD)	Mean	(SD)				
Mean number of IEC channels received from the program	3.04	(1.2)	3.25	(1.6)	0.179* ^3^			0.164* ^4^

* 1: Chi-square test or Fisher's exact test.

* 2: Logistic regression analysis.

* 3: Independent-sample t-test.

* 4: Hierarchical multiple regression analysis.

Adjusted for caregiver type, age, occupation, and education level; number of people in household; refrigerator possession; number of children under five years; child's birth order, age and sex; WTF water access level; and main water source for drinking, cooking, food preparation, and laundry and bathing.

### Diarrhea prevalence among children

In the baseline survey, 21.6% of caregivers reported that their child had experienced diarrhea during the previous two weeks (Table 4). The childhood diarrhea prevalence was significantly reduced to 7.6% at the 1^st^ evaluation (P = 0.002) – a reduction that was maintained through the 2^nd^ evaluation (5.9%). Stratifying diarrhea prevalence by monthly categories showed that diarrhea tended to be more prevalent among children under 24 months than among older children. Prevalence of diarrhea was largely reduced, particularly among children older than 24 months, by the 2^nd^ evaluation. Considering that the highest proportion of children was under 24 months at baseline and at the 1^st^ evaluation relative to the 2^nd^ evaluation, we adjusted for child's age along with other confounding factors, but the results were essentially the same as in the unadjusted analysis.

**Table 4:** Diarrhea prevalence among children under 5 years.

	Baseline		1^st^ evaluation		2^nd^ evaluation		Baseline to 1^st^ evaluation				1^st^ evaluation to 2^nd^ evaluation			
	n/N	(%)	n/N	(%)	n/N	(%)	P value* ^1^	AOR	(95% C.I.)	Adjusted P value* ^2^	P value* ^1^	AOR	(95% C.I.)	Adjusted P value* ^2^
**Diarrhea prevalence in the past two weeks**														
Under 5 years	27/125	(21.6)	10/132	(7.6)	11/185	(5.9)	0.001	0.22	(0.08–0.57)	0.002	0.565	1.26	(0.38–4.19)	0.701
**Stratified by month category**														
6–11 months	4/18	(22.2)	2/13	(15.4)	2/21	(9.5)								
12–23 months	10/35	(28.6)	5/39	(12.8)	8/38	(21.1)								
24–35 months	7/27	(25.9)	1/39	(2.6)	0/42	(0.0)								
36–47 months	4/23	(17.4)	2/23	(8.7)	1/49	(2.0)								
48–59 months	2/22	(9.1)	0/18	(0.0)	0/35	(0.0)								

* 1: Chi-square test.

* 2: Logistic regression analysis.

Adjusted for caregiver type, age, occupation, and education level; number of people in household; refrigerator possession; number of children under five years; child's birth order, age, and sex; WTF water access level; and main water source for drinking, cooking, food preparation, and laundry and bathing.

### Changes in FHFS behaviors

Overall, among 17 FHFS behaviors measured, a total of 11 behaviors were improved or maintained by the time of the 2^nd^ evaluation (Table 5). Handwashing after using the toilet significantly improved both from baseline to the 1^st^ evaluation (from 22.0% to 33.3%, P = 0.001), and from the 1^st^ to the 2^nd^ evaluation (from 33.3% to 53.8%, P = 0.002). Notably, all three behaviors that improved from baseline to the 1^st^ evaluation were related to food safety. Adherence rates ranged from 61.6% to 87.9% at baseline, and showed an absolute change of 9.1% to 35.3% by the 1^st^ evaluation – a level maintained at the 2^nd^ evaluation. In contrast, all seven behaviors that improved from the 1^st^ to the 2^nd^ evaluation were related to food hygiene. Adherence rates were lower (8.1% to 42.0%) than food safety behaviors at baseline, but significant improvements (12.0% to 25.5%) were observed by the 2^nd^ evaluation. The adherence rate for handwashing after handling garbage, however, significantly decreased from the 1^st^ to the 2^nd^ evaluation. Five FHFS behaviors did not significantly improve either from baseline to the 1^st^ evaluation or from the 1^st^ to the 2^nd^ evaluation. However, four of these behaviors showed an absolute change from 4.5% to 11.0%, and one reached 100% adherence by the 2^nd^ evaluation.

**Table 5:** FHFS behaviors.

	Baseline			1^st^ evaluation			2^nd^ evaluation		Baseline to 1^st^ evaluation				1^st^ evaluation to 2^nd^ evaluation					
	n/N	(%)		n/N		(%)	n/N	(%)	Absolute change%	P value* ^1^	Adjusted P value* ^2^		Absolute change%			P value* ^1^	Adjusted P value* ^2^	
**Caregivers' food hygiene behaviors**																		
**Handwashing with soap at food eating and handling-related points**																		
** Before eating**																		
34/125		(27.2)		33/132		(25.0)	93/184	(50.5)	−2.2	0.688	0.762		25.5			P<0.001	P<0.001	
** Before feeding**																		
26/125		(20.8)		27/132		(20.5)	84/184	(45.7)	−0.3	0.945	0.718		25.2			P<0.001	P<0.001	
** Before food preparation**																		
24/112		(19.2)		25/132		(18.9)	67/184	(36.4)	−0.3	0.958	0.131		17.5			0.001	0.001	
** After handling raw food**																		
10/124		(8.1)		12/132		(9.1)	39/185	(21.1)	1.0	0.770	0.632		12.0			0.004	0.005	
**Handwashing with soap at sanitation-related points**																		
** After using the toilet**																		
27/123		(22.0)		44/132		(33.3)	99/184	(53.8)	11.3	0.043	0.001		20.5			P<0.001	0.002	
** After cleaning child's bottom**																		
21/124		(16.9)		24/132		(18.2)	42/185	(22.7)	1.3	0.793	0.536		4.5			0.328	0.290	
** After handling garbage**																		
13/124		(10.5)		16/132		(12.1)	13/184	(7.1)	1.6	0.680	0.532		−5.0			0.125	0.015	
** When hands look dirty**																		
15/122		(12.3)		22/132		(16.7)	70/184	(38.0)	4.4	0.324	0.552		21.3			P<0.001	P<0.001	
**Children's food hygiene behaviors**																		
** Handwashing with soap before eating**																		
40/119		(33.6)		44/132		(33.3)	90/185	(48.6)	−0.3	0.963	0.881		15.3			0.007	0.020	
** Handwashing with soap after toilet use**																		
47/112		(42.0)		51/123		(41.5)	102/184	(55.4)	−0.5	0.938	0.984		13.9			0.016	0.037	
**Caregivers' food safety behaviors**																		
**Cross-contamination**																		
** Using separate utensils (cutting board and knife) for raw food and cooked food**																		
77/125		(61.6)	110/132		(83.3)		152/185	(82.2)	21.7	<0.001		<0.001		−1.1	0.786		0.682	
** Washing child's utensils (cup, bowl and spoon) with soap**																		
109/124		(87.9)	128/132		(97.0)		181/185	(97.8)	9.1	0.006		0.008		0.8	0.627		0.718	
** Food preparation on tables**																		
87/125		(69.6)	86/132		(65.2)		141/185	(76.2)	−4.4	0.447		0.618		11.0	0.031		0.096	
**Food at safe temperature**																		
** Good cooked food storage behavior (food for child in summer, more than 2 hours)**																		
88/125		(70.4)	107/132		(81.1)		162/185	(87.6)	10.7	0.046		0.125		6.5	0.111		0.317	
** Good cooked food storage behavior (food for child in summer, less than 2 hours)** * **^3^**																		
79/125		(63.2)	130/132		(98.5)		185/185	(100.0)	35.3	<0.001		<0.001		1.5	0.093		0.999	
** Good raw food storage behavior (food for child in summer)** * **^4^**																		
125/125		(100.0)	129/130		(99.2)		183/183	(100.0)	−0.8	0.326		0.999		0.8	0.235		1.000	
**Adequate cooking**																		
** Reheat leftovers of whole family before eating (food for whole family)** * **^5^**																		
––		––	121/128		(94.5)		179/182	(98.4)		–––		–––		3.9	0.061		0.068	
**FHFS score (out of 14 items)**																		
N		Mean (SD)	N		Mean (SD)		N	Mean (SD)										
107		4.96 (2.9)	123		5.50 (2.3)		183	7.23 (2.4)		0.129* ^6^		0.047* ^7^			<0.001* ^6^		<0.001* ^7^	

* 1: Chi-square test or Fisher's exact test.

* 2: Logistic regression analysis.

* 3: Excluded from the FHFS score because none of the caregivers reported wrong cooked food storage behavior at the 2nd evaluation.

* 4: Excluded from the FHFS score because none of the caregivers reported wrong raw food storage at baseline and at the 2nd evaluation.

* 5: Excluded from the FHFS score because we did not measure this indicator in the baseline survey.

* 6: Independent-sample t-test.

* 7: Hierarchical multiple regression analysis.

Adjusted for caregiver type, age, occupation, and education level; number of people in household; refrigerator possession; number of children under five years; child's birth order, age, and sex; WTF water access level; and main water source for drinking, cooking, food preparation, and laundry and bathing.

### Factors linked to having a greater number of good FHFS behaviors at the 2^nd^ evaluation

Important factors found to be related to having a greater number of good FHFS behaviors through the 2^nd^ follow-up evaluation were possession of a refrigerator (Beta = 0.147, p =  0.046) and greater access to the WTF water (Beta = 0.149, P = 0.041) (Table 6). Among the program's multiple IEC channels, flip chart communication (Beta = 0.174, P = 0.018) by a community group was significantly associated with presence of a greater number of good FHFS behaviors.

**Table 6:** Determinants of the number of good FHFS behaviors at the 2^nd^ evaluation.

	Beta coefficient	SE	t	P value* ^1^
**Use of multiple IEC channels (n = 183)** * **^2^**				
Refrigerator possession	0.147	0.427	2.01	0.046
Number of children under five years	0.121	0.479	1.66	0.099
WTF water access level	0.149	0.382	2.06	0.041
Multiple IEC channels from the project (scored from 1 to 5)* ^3^	0.131	0.109	1.81	0.072
**Effective IEC channel (n = 183)** * **^4^**				
Refrigerator possession	0.127	0.422	1.76	0.081
WTF water access level	0.172	0.385	2.36	0.019
Received flip chart communication	0.174	0.352	2.39	0.018

* 1: Multiple linear regression with backward elimination procedures.

* 2: The first model included 10 socio-demographic factors, 5 water use factors and multiple IEC channels from the program. The final model included variables for which P values were less than 0.1.

* 3: Continuous variable.

* 4: The first model included 10 socio-demographic factors, 5 water use factors, 3 mass media channels and 5 IEC channels from the program. The final model included variables for which P values were less than 0.1.

## Discussion

This study clearly demonstrates that 11 FHFS behaviors improved or were maintained when a WMU conducted IEC activities through a self-sustaining program. WMU-administered flip chart communication emerged as the most effective IEC channel for improving the greatest number of FHFS behaviors.

The improvement of multiple behaviors encompassing both FHFS disciplines suggests that multiple behaviors can be changed without reducing the total number of critical behaviors when these behaviors are decided based on group discussions with caregivers. Conventional methods, targeting only a small number of behaviors directly related to reductions in the incidence of diarrhea, can be suitable for showing outcomes in short-term studies [12]. However, multiple behavior change is necessary when aiming to change FHFS-related community social norms associated with diarrhea incidence reduction. Multiple behavior change has also been demonstrated in a previous study in which handwashing and other sanitation-related behaviors were combined toward building on community needs [19]. This study provides new results to demonstrate that multiple FHFS-related behaviors can be improved when these priorities are established through group discussions.

In the present study, caregivers were provided with many messages related to each targeted behavior in order to promote improvement of a great number of FHFS behaviors [32], whereas previous methods have focused on only a few messages [12]. This method was found to be acceptable and affordable to caregivers in our study for several reasons. First, at least some caregivers were practicing the targeted behaviors even prior to the intervention [33]. Hence, even though many messages were provided, caregivers might have only selected and acted upon behaviors that had not been practiced previously. Further, several behaviors were found to be practiced at slightly higher frequencies than the national average [34]. Regarding affordability, an increase in the number of households engaging in home-based businesses may lead to better financial situations and thus provide improved opportunities to purchase cooking equipment and soap [14].

Our results indicate that a lower adherence rate at baseline may require a longer period of time (1 year) to achieve certain improvements. This is in line with a study in Burkina Faso that similarly highlighted the length of time required to effect substantial behavior change [14]. Community-based IEC activities could be one effective method to implement IEC activities in the long term, toward which this study indicates two useful steps to be taken. First, the formation of community groups helps to sustain IEC activities [35]. Second, providing IEC training to community groups enhances promotion of good FHFS behaviors at the grassroots level in the long term [10]. In this way, the cost of long-term IEC activities can be minimized [36].

When we examined the impacts of the different IEC channels, flip chart communication emerged as the most effective IEC channel to improve the greatest number of FHFS behaviors. Flip chart communication during household visits had a number of advantages for both IEC providers and caregivers in this study. For IEC providers, flip chart communication functions as a practical guide when communicating with caregivers, allowing them to deliver complete messages without missing any elements. Also, since flip charts are portable, they are easy for IEC providers to handle when visiting homes. Moreover, flip charts appear to be attractive to caregivers because of the easy-to-understand, colorful images. Compared to other communication channels, interpersonal communication expedites the exchange of information between caregivers and IEC providers and deepens caregivers' understanding of the benefits of good FHFS behaviors in accordance with their preparedness [33]. These factors may contribute to more frequent handwashing behaviors and proper food handling practices among caregivers. In this regard, sustainable IEC activities in Vietnam should include features to improve interpersonal communication using portable IEC materials.

Demonstrated advantages notwithstanding, our study also indicates that careful consideration is needed to overcome the disadvantages of flip chart communication. Household visits entail continuous effort and manpower in areas where population density is low [15], [37]. This burden may be reduced if several households could be gathered in one place to conduct the flip chart communication. Additionally, the training of IEC providers may influence the effectiveness of this channel. This dimension can be improved by including role-play sessions in the training. Role-play exercises allow IEC providers the opportunity to practice sharing their personal experiences with good FHFS behaviors and connecting such behaviors with reduction in diarrhea incidence. Such experiences may play an important role in communicating with caregivers where strong community relationships are in place [32].

These findings should be considered in the context of several study limitations. First, self-reporting measurements may come with an overestimate of health outcomes and behaviors due to recall bias [38]. Although direct observation and recording methods could conceivably have been combined, we concluded that considerations such as the potential intrusiveness and inconvenience of such methods were more important, especially in a long-term community-based study [11]. Therefore, we relied solely on a follow-up focus group discussion to retrospectively analyze how the behavior change had happened at both the individual and societal levels. In Vietnam, although a difference between knowledge and practice has been noted in a previous handwashing program [39], reliable and feasible data collection tools are not currently available. More research is thus needed to develop innovative tools to minimize bias in measuring FHFS behaviors [11]. Second, this study did not include control sites due to ethical concerns and practical considerations [40]. Although we could not disregard the potential influence from other regional or national programs, we confirmed through monthly monitoring reports that FHFS information provided through mass media remained relatively unchanged over the 2-year period. Third, this study was conducted in one village in suburban Hanoi, and differences in geographic location were not assessed. Similar IEC methods should be replicated in a medium- to-large-scale program in rural and remote areas to confirm the effectiveness in such settings.

## Conclusions

This study found that, when community groups conducted IEC activities, multiple behaviors that combine FHFS elements were improved. This positive finding is largely due to an innovative method that sets multiple target behaviors through group discussions with caregivers and provides practical messages to guide each behavior. For FHFS behaviors for which the adherence rate is low, long-term IEC activities rooted in the community are necessary to effect improvements. As this study found, flip chart communication by household visits could be an effective tool for both IEC providers and caregivers in this vein. For IEC providers, flip charts facilitate comprehensive delivery of many messages simultaneously, minimizing missed information. For caregivers, colorful and easy-to-understand images are interesting, and flip chart communication offers an opportunity to learn a wide range of good FHFS behaviors. Toward sustainable IEC activities to improve multiple FHFS behaviors linked to reduced childhood diarrhea incidence, interpersonal communication using portable materials, such as flip chart communication by a community group, is thus recommended for inclusion in water and health programs in Vietnam.

## Supporting Information

File S1 **Supplementary research proposal.**
(DOC)
