# Supplementary material for: Long-Term Impact of Community-Based Information, Education and Communication Activities on Food Hygiene and Food Safety Behaviors in Vietnam: A Longitudinal Study
Source: PLoS One. 2013 Aug 12;8(8):e70654. doi: 10.1371/journal.pone.0070654 (PMC3741207; doi:10.1371/journal.pone.0070654)
Supplement: File S1 — Supplementary research proposal. (DOC) [file pone.0070654.s001.doc]

**Research Proposal**

**Evaluation of a safe water supply, nutrition and health education program in Vietnam**

# International Life Sciences Institute Japan Center for Health Promotion

# National Institute of Nutrition, Vietnam

Department of Community and Global Health, The University of Tokyo

**Introduction**

**Brief description of the project**

The prevalence of malnutrition among women and children remains high in Vietnam. The cause of malnutrition is complex, but hygiene practices is an important underlying cause of malnutrition. Clean water is primary health factor and is the first priority of public health. Clean water also reflects the quality of life. Only about 25%-30% of the population in Vietnam has access to “clean water”, based on the standards established by the Vietnamese Government.

Since 2001, the International Life Sciences Institute Japan Center for Health Promotion (ILSI CHP Japan) has monitored the quality of drinking water from 16 sources in rural areas in cooperation with the National Institute of Nutrition (NIN), Ministry of Health (MoH) and Ministry of Agriculture and Rural Development (MARD). The outcome of this monitoring shows that the quality of more than a half of water sources analyzed display cause for concern. Additionally, a survey on the needs of communities, that was carried out in 2004 showed that people have a strong willingness to participate in activities aimed at improving water quality and the health and nutrition status of people in the community. Furthermore, a study which simulated the water treatment system indicated that water quality could be improved to meet the Vietnamese standards by optimizing the operations of the existing water treatment facilities.

To overcome above situation, we developed a project “Improvement of the Nutritional Status of Women and Children through a Participatory Approach for Safe Water in Rural Communes in Vietnam”. The aims of the project include the following: 1) improving the knowledge and practices related to clean water and hygiene by community and household members; 2) improving the quality of drinking water available to families to a level which meets Vietnamese standards and which can be sustained, 3) improving the health and nutritional status of children and women.

At present, we know of no such effective and sustainable program aimed at ensuring safe water supplies through participatory approach and aimed at attaining better nutrition for women and children.

Since this proposed project follows up on the water quality monitoring program conducted by ILSI Japan CHP since 2001, ILSI Japan CHP has proposed a JICA Partnership Program, Grass-roots Partner Project, aimed at improving the safe water supply and health and hygiene conditions and therefore attain better nutrition for children and women.

**Justification**

Over the past 5 years, Vietnam has achieved remarkable results in reducing the childhood malnutrition rate. However, the prevalence of malnutrition remains very high*.* By the year 2004, prevalence of malnutrition in children under 5 was 26.6% underweight and 30% stunting. The malnutrition rate is higher in rural areas. More than 30% of women of reproductive age suffer from Chronic Energy Deficiency (CED) and recently, a NIN study on lactating mothers showed a CED rate of 38.7%. In coming years, it will not easy to achieve the same success rate as we have seen in recent years. The challenges are poverty and food insecurity, fewer opportunities for improvements related to malnutrition, poor environmental conditions for young children for which safe water plays an important role and diarrhoeal diseases linked to poor water quality and hygiene condition that can lead to malnutrition in children, especially stunting.

One of the first priorities for public health is clean water. Clean water also reflects the quality of life. Recently, many experts have proposed the rate of people who utilize clean water as an indicator for the health environment. According to the International Food Policy Research Institute (IFPRI, 2/2000) improvements in the clean water indicator is usually closely related to improved health care. When the number of people who utilize clean water increases, the infectious disease rate, a factor directly related to child malnutrition, is reduced. Water and environmental sanitation also affect food hygiene and human sanitation practices. As the result, the efforts of Government to ensure a safe water supply and environmental sanitation positively influence the nutritional and health status of children.

In recent years, the Vietnamese government has strongly supported improvements aimed at improving the availability of safe water. The percentage of the population with access to safe water increased from 12% in 1990 to 18% in 1992. More recently, a report from the Safe Water Program, Ministry of Agriculture and Rural Development (1999) showed that actually 60% of the urban population and 30% of the rural population have access to safe water. So the rate of access to safe water in rural areas is still low and requires more investment in coming years. In addition, the percentage of the population having access to proper latrines differs greatly by region. In the Mekong river delta area, only 42.9% of households do not have a proper latrine. The percentage of the population in Nam Dinh which utilizes proper hygienic latrines is low, and there is very little distance from latrines to water wells ( <10 m), which is a risk for water contamination. Therefore, promotion of safe water and environmental sanitation in rural areas is important for improving living conditions and the nutritional status of the target population.

According to the objectives of the National Program for Clean Water under the Ministry of Agriculture and Rural Development, which was conducted until 2005, 80% of the rural population should utilize clean water for food and living activities, and 50% of the population should use properly hygienic latrines. In a 2003 survey, The Department of Preventive Medicine (MoH) in Nam Dinh province reported that the sources for drinking water were rain water (87.1%), deep well and drill well water. In addition, many houldholds utilize water from ponds and rivers. In 2001, ILSI Japan CHP conducted quality monitoring of drinking water from 16 water sources in rural areas in cooperation with the National Institute of Nutrition (NIN), Ministry of Health (MoH) and Ministry of Agriculture and Rural Development (MARD). The outcome shows cause for concern about the quality of more than half the water sources analyzed. There are no plans for city water supply systems to be established in rural areas for anytime in the near future.

In conjunction with improvements in water supply systems, IEC activities are very important in promoting the role of clean water and nutrition and health status of women and children. Some IEC programs have been developed and implemented in rural provinces but the effect has not been measurable. A 2003 survey by the Department of Preventive Medicine (MoH) in Nam Dinh province showed that 90.4% of the inhabitants wanted clean water, and 96.7% of these desired piped water. However, only 84.5% of inhabitants actually registered for using the piped water. IEC activities would be vital to increasing the number of people with access to clean and piped water. The Project "Improvement of Nutritional Status in Women and Children Through a Participatory Approach for Safe Water in Rural Communes in Vietnam**”** would be very important at this time.

**Objectives**

**Long-term objective**

To establish a sustainable water supplies and health related communication model in rural areas through a participatory approach

**Specific objectives**

- To improve the awareness of and the practices related to clean water, food hygiene, food safety and environment sanitation by community and household members.
- To improve the quality of drinking water to meet Vietnamese standards for families in a way that can easily be sustained.
- To improve the health and nutritional status of women and children.

**Methods and materials**

**Research design**

This is a repeated, cross-sectional study.

**Study site**

- Tam Hiep Commune, Thanh Tri District, Hanoi
- Dai Mo Commune, Tu Liem District, Hanoi
- Quang Trung Commune, Vu Van District, Nam Dinh Province

**Participants**

Participants will be caregivers with children from 6 months to 4 years old. Participants will be invited to join the study based on the list of children under 5 years old kept in the commune health centers.

**Sample size**

Sample size will be calculated with confidence level of 95% and a power of 80%. We expect to detect a 25% of difference in the percentage of healthy behaviours from the baseline to the evaluation. In each commune, a minimum of 120 caregiver-child pairs will be interviewed.

**Measurement**

Outcome variables will be i) episodes of diarrhoea in the past 2 weeks (Yes or No) and ii) the hygiene practice of caregivers. Independent variables will be socio-economic status and the water use details.

We will develop a questionnaire which will be composed of: i) general information, ii) nutrition and health, iii) food hygiene and safety, iv) water management data, and v) child anthropometric measurement and clinical examinations.

The questionnaire will be developed based on the existing questionnaire in Vietnam with reference to WHO/UNICEF guidelines. The English version will be developed and translated into Vietnamese, and finally back into English. A pilot test will be conducted with 25 caregivers in a non-study commune to confirm the caregivers are able to understand the meaning of the questionnaire.

**Data collection**

Data collection will be carried out at the baseline, at one year into the study, and at two years into the study.

We will hire 8 staff from the NIN to obtain answers from caregivers via the questionnaire. The interview should take about 40 minutes and the anthropometric measurements should take about 5 minutes.

**Data analysis**

Data analysis will be conducted using SPSS for Windows.

**Ethical considerations**

Health volunteers will explain the purpose and the objective of the study using the information sheet, and an informed consent sheet will be distributed.

Ethical approvals will be obtained from the Research Ethics Committee of the Graduate School of Medicine of the University of Tokyo, Japan. The ethical approval will also be obtained from the Ethical Committee of the National Institute of Nutrition, Vietnam.
